# Multi-Scale Spatiotemporal Graph Neural Network Using Brain Partitioning for Major Depressive Disorder Detection

**DOI:** 10.3390/s26092868

**Published:** 2026-05-04

**Authors:** Zhao Geng, Wei Guo, Jiale Wang, Yonghua Ma, Yongbao Zhu

**Affiliations:** 1 School of Public Health, Shandong Second Medical University, Weifang 261053, China; 2 National Administration of Health Data, Jinan 250002, China

**Keywords:** major depressive disorder (MDD), electroencephalogram (EEG), graph neural network (GNN), hemispheric partitioning

## Abstract

Major depressive disorder (MDD) is a prevalent and severe mental disorder, and EEG-based automated detection has become a promising approach for auxiliary screening diagnosis. In this work, we propose a novel multiscale spatiotemporal graph neural network for MDD detection from multichannel EEG signals. Specifically, a left–right hemispheric partitioning prior is used to encode brain functional organization. Based on this partitioning, adaptive graphs are then constructed and graph message passing is performed to model intra-hemispheric interactions. The approach not only incorporates brain functional organization into the learning process but also enhances the extraction of discriminative features related to depressive brain dynamics. The proposed method was validated in a cross-subject scenario on a private resting-state EEG dataset including 54 adult participants (27 MDD patients and 27 healthy controls; age range: 27–48 years). Experimental results on the dataset achieve an accuracy of 92.21%, surpassing the baseline models. Meanwhile, ablation experiments demonstrate the effectiveness of our proposed method.

## 1. Introduction

Major depressive disorder (MDD) is a prevalent mental illness that affects people of all ages worldwide and has been recognized by the World Health Organization (WHO) as one of the leading causes of disability [1,2]. A 2023 WHO report estimates that approximately 280 million people are living with depression. Unlike transient mood fluctuations in daily life, MDD is a persistent and debilitating condition that can lead to severe functional impairment and a range of adverse health outcomes. It is also associated with an elevated risk of suicide compared with healthy individuals [3]. In addition, MDD places a substantial economic burden on both individuals and society. Therefore, accurate and timely detection of MDD is crucial for both scientific research and societal well-being.

Currently, the primary methods for diagnosing depression rely on clinician–patient communication and psychiatric questionnaire assessments [4]. However, their accuracy is influenced by clinicians’ experience and may be compromised by subjective bias, denial, or symptom concealment on the part of patients. Moreover, MDD is characterized by heterogeneous clinical presentations [5], and patients often exhibit variable and sometimes unpredictable responses to standard pharmacological and psychotherapeutic interventions [6], which further complicates diagnosis, treatment planning, and outcome prediction. Consequently, developing objective assessment approaches that do not depend solely on complex clinical judgments has become an active research focus [7]. In this context, electrophysiological and neurophysiological signals have attracted increasing attention for MDD analysis, including functional magnetic resonance imaging (fMRI) [8,9], electrocardiogram (ECG) [10,11], and electroencephalogram (EEG) [12,13]. Among these modalities, EEG is particularly appealing due to its ease of acquisition, low cost, non-invasiveness, and high temporal resolution.

To date, many EEG-based methods for brain state detection have been developed. Early studies combine manually extracted EEG features with machine learning classifiers [14,15]. For example, Sallum et al. [16] constructed functional connectivity networks using the phase lag index and applied a support vector machine to detect depression. Ahmed et al. [17] proposed a stacked ensemble machine-learning framework for depression identification. However, the inherent nonlinearity and inter-individual variability of EEG signals call for further improvements in detection performance. In recent years, the development of deep learning has led to its widespread application in analyzing brain states [18,19]. For example, Khan et al. [20] proposed a 2D-CNN model using wavelet coherence to detect MDD. Deng et al. [21] developed an attention-based SparNet to capture spatial- and frequency-domain patterns from both local brain regions and the whole brain for depression detection. Chen et al. [22] introduced a self-attention graph pooling strategy and incorporated prior knowledge by adding global connections in the adjacency matrix to improve depression detection.

Despite the progress described above, most existing EEG-based approaches mainly emphasize temporal and spectral representations while often overlooking the coordinated activity among brain regions. Brain function emerges from interactions within and across distributed regions rather than isolated channel-wise patterns. Many studies have shown that neurological and psychiatric disorders are associated with abnormal brain functional topology [23]. Graph-based learning provides a natural way to represent EEG channels or brain regions as nodes and their interactions as edges, thereby enabling explicit modeling of coordinated dynamics. Specifically, graph convolutional networks (GCNs) can perform message passing over the constructed topology, effectively capturing the complex dependency patterns among regions [24,25]. For example, Zhu et al. [26] proposed an improved GCN that enhances the representation of key nodes and connections for depression detection. Li et al. [27] used a GCN framework that leverages fine-grained EEG signals and graph mutual information maximization for depression recognition. Zhang et al. [28] combined subject-level partitioning with GCN for depression detection. These studies demonstrate the potential of graph-based modeling for EEG-based depression analysis and also indicate that the way EEG channels are organized in graph construction plays an important role in model performance.

In this work, we further develop graph-based EEG modeling from the perspective of hemispheric organization, motivated by previous studies reporting hemispheric asymmetry and lateralized abnormalities in MDD-related brain activity [29,30]. On this basis, hemispheric partitioning is introduced into EEG graph learning and further integrated with multiscale temporal modeling and adaptive region-level fusion in a unified framework. Based on this design, we propose a framework called MSBP-GNN for recognizing MDD from multi-channel EEG data. It consists of three key components: a multi-scale temporal filtering module (MSTF module), a brain-partition-based graph learning module (BPGL module), and an attentive fusion classification module (AFC module). The main contributions of this work are summarized as follows:

(1) The MSTF module extracts temporal patterns from raw EEG signals by utilizing parallel temporal convolutions with varying receptive fields, allowing the model to capture different temporal features from the signals effectively.
(2) The BPGL module introduces hemispheric partitioning as a physiologically inspired structural prior for EEG graph learning, applying graph convolutions within predefined brain regions to model intra-region dependencies in a more structured manner.
(3) The AFC module adaptively aggregates region-level features and uses an attention mechanism to fuse information across regions, enhancing MDD detection by capturing both intra-region and inter-region dependencies.

The remainder of this paper is organized as follows. Section 2 introduces the dataset and describes the preprocessing procedures. Section 3 details the proposed MSBP-GNN framework and its core modules. Section 4 presents the experimental setup and a comprehensive analysis of the experimental results. Finally, Section 5 concludes the paper and discusses potential future work.

## 2. Dataset Description

### 2.1. Dataset Acquisition

The dataset was recorded at the Affiliated Hospital of Shandong University of Traditional Chinese Medicine (Shandong, China). A total of 54 adult participants were enrolled, consisting of 27 patients diagnosed with major depressive disorder (MDD) and 27 age-matched healthy controls (HCs). All MDD patients were diagnosed by senior psychiatrists according to the ICD-10 criteria for moderate-to-severe unipolar depression without psychotic symptoms. In this study, only MDD patients without psychotic symptoms were included in order to reduce clinical heterogeneity and limit potential confounding effects of psychotic features on EEG patterns, thereby allowing a more focused evaluation of the proposed MDD detection framework. Patients with any comorbid psychiatric conditions apart from personality disorders, or with any neurological or somatic diagnoses apart from hypertension and hyperlipidemia, were excluded. Both the MDD patients and healthy participants were volunteers and signed the informed consent form for participation. The ethics committee of the hospital approved the experimental design, which was explained to all participants. The healthy participants were examined for clinical symptoms to exclude the possibility of any physical or mental disorder and were confirmed to be normal. Prior to EEG recordings, participants were instructed to abstain from coffee, nicotine, and alcohol. In addition, to reduce medication-related effects on EEG patterns, the MDD patients underwent a two-week washout period before the first EEG recording, and only the pre-treatment EEG data were used in this study. To ensure consistency, all recordings were conducted at the same time of day for all participants.

The subjects were matched for age and sex as closely as possible; nevertheless, minor discrepancies remained. Detailed demographic information for all participants is summarized in Table 1. The overall mean age was 35.70 ± 4.49 years in the HC group and 37.15 ± 5.37 years in the MDD group, and no significant between-group age difference was observed (p=0.289).

**Figure 1:**
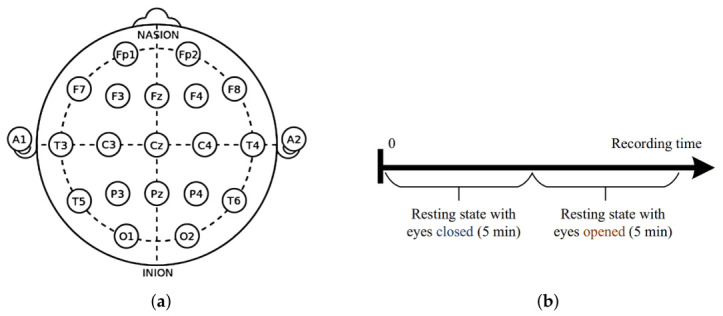
(**a**) The 10–20 system with 19 channels. (**b**) EEG recording protocol.

The experimental data acquisition included clinical scores and EEG data. The clinical data involved questionnaires for the assessment of disease severity, such as the Beck Depression Inventory-II (BDI-II) and the Hospital Anxiety and Depression Scale (HADS) [31,32]. As shown in Figure 1a, the EEG data were recorded using a 19-channel EEG amplifier with a standard 10–20 electrode array [33] with the linked ear (LE) as a reference, covering the frontal (Fp1, Fp2, F3, F4, F7, F8, Fpz); temporal (T3, T4, T5, T6); parietal (P3, P4, P7, P8); occipital (O1, O2); and central (C3, C4) regions. Additionally, the sampling frequency was 256 Hz. The experimental paradigm consisted of a resting-state recording, including 5 min of eyes-closed (EC) and 5 min of eyes-open (EO) conditions (Figure 1b). During the session, subjects remained in a comfortable sitting-down position in a quiet and peaceful room to minimize physiological artifacts. A trained technician monitored the entire recording session, marking significant events and interference such as eye blinking, swallowing, or body movements to ensure the integrity of the data for later preprocessing.

### 2.2. Preprocessing

**Figure 2:**
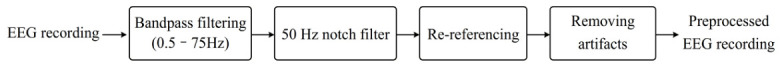
EEG preprocessing protocol.

Raw EEG signals are inevitably contaminated by various types of noise and physiological artifacts, and may not accurately reflect the underlying brain activity. Therefore, EEG preprocessing was necessary to improve signal quality and to obtain cleaner neural activity for subsequent analysis. The preprocessing was performed using the Matlab R2021b version EEGLAB toolbox. As illustrated in Figure 2, the preprocessing protocol consisted of four main steps. The raw EEG signals were first filtered using a band-pass filter between 0.5 and 70 Hz to suppress slow trends and high-frequency interference while preserving the frequency components of interest. Then, a 50 Hz notch filter was applied to remove power-line noise. Third, the signals were re-referenced to an infinity reference to provide a standardized reference scheme for analysis [34]. Finally, independent component analysis (ICA) was conducted to decompose the EEG into independent components, and artifact-related components corresponding to eye activity, muscle activity, and other non-neural sources were identified and removed, resulting in the preprocessed EEG recordings used in the experiments.

## 3. Methods

**Figure 3:**
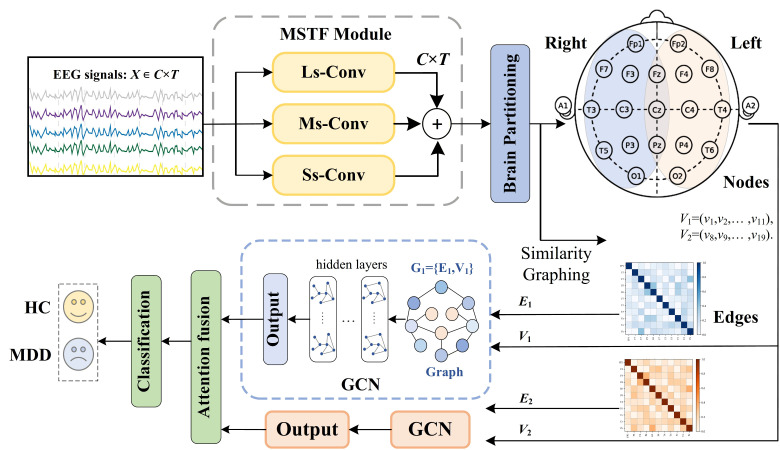
The architecture of the proposed MSBP-GNN.

In this section, we introduce the design concept and overall architecture of MSBP-GNN. As illustrated in Figure 3, MSBP-GNN is built upon the idea of jointly modeling temporal dynamics and partition-aware spatial interactions in a multi-channel EEG. It includes three main modules: an MSTF module for temporal feature extraction, a BPGL module that groups electrodes into predefined regions to extract intra-region features and an AFC module that integrates inter-region features for MDD detection.

### 3.1. Multi-Scale Temporal Filtering Module

EEG signals contain rich time–frequency fluctuations and exhibit pronounced multi-band and multi-scale characteristics. To better capture temporal features and improve representation robustness, we design a multi-scale temporal filtering module with three parallel depthwise 1D convolutional branches.

Given an input sample $X\in R^{C\times T}$, each branch performs channel-wise temporal filtering via grouped depthwise convolution with groups=C, followed by batch normalization and ELU activation. The three branches use kernel lengths corresponding to temporal windows of 0.4 s, 0.2 s, and 0.1 s, respectively. Specifically, the kernel length for branch *m* is computed as

(1) $$k_{m}=odd\left(\alpha_{m}f_{s}\right),\;\alpha_{m}\in \left\{0.4,0.2,0.1\right\},$$

where $f_{s}$ is the sampling rate and odd(·) enforces an odd kernel size to enable the same padding and preserve the temporal length.

For each branch m∈{1,2,3}, the filtered feature map is computed as

(2) $$X_{m}=\sigma \;\left(BN\;\left(DWConv_{k_{m}}\left(X\right)\right)\right),\;X_{m}\in R^{C\times T},$$

where $DWConv_{k_{m}}\left(\cdot \right)$ denotes depthwise 1D convolution with kernel length $k_{m}$, BN(·) is batch normalization, and σ(·) is the ELU activation.

Finally, we aggregate the multi-scale responses by mean fusion to obtain a unified temporal representation:

(3) $$X^{\left(1\right)}=\frac{1}{3}\sum_{m=1}^{3}X_{m},\;X^{\left(1\right)}\in R^{C\times T}.$$

By explicitly modeling temporal receptive-field diversity, this module enhances the model’s sensitivity to frequency-dependent patterns and provides a robust input for subsequent region-wise graph construction and node-level temporal embedding. More specific parameters for MSTF module are detailed in Table 2.

### 3.2. Brain-Partition-Based Graph Learning Module

#### 3.2.1. Regional Sparse Adjacency Construction

Brain activity is inherently localized to specific regions of the brain, with each region performing distinct functional roles. To capture these region-specific dynamics and better model the interactions within each region, we partition the 19 EEG channels based on anatomical priors into distinct brain regions. There are different brain partition strategies, such as two-region partitioning (left and right hemispheres), anterior–posterior partitioning (frontal and parietal regions), and four-region partitioning that combines both hemispheric and anterior–posterior divisions. In this work, we selected the left–right hemispheric partitioning strategy after comparison. Specifically, the left hemisphere includes electrodes FP1, F7, F3, Fz, T3, C3, Cz, T5, P3, Pz, and O1, while the right hemisphere includes FP2, F8, F4, Fz, T4, C4, Cz, T6, P4, Pz, and O2. Furthermore, the midline channels (e.g., Fz, Cz, Pz) are shared between by both hemispheric graphs due to their central role in both lateralized and bilateral brain functions. This treatment allows the model to preserve shared information around the hemispheric boundary and provides a more complete description of hemisphere-related functional interactions.

This partitioning strategy allows the model to focus on intra-region interactions and enhances the sensitivity to functional patterns specific to each hemisphere, capturing more precise and region-dependent brain activity. Building upon this region-level partitioning, we construct sparse functional graphs within each hemisphere to model structured intra-regional interactions. For each region i∈{l,r} (left and right hemispheres), we compute the pairwise cosine similarity between L2-normalized temporal sequences to capture their functional connectivity. Specifically, normalization is performed as

(4) $$\hat{X_{i}^{\left(1\right)}}\left(j,:\right)=\frac{X_{i}^{\left(1\right)}\left(j,:\right)}{∥X_{i}^{\left(1\right)}{\left(j,:\right)∥}_{2}},$$

and the adjacency matrix is computed as

(5) $$A_{i}=Top\text{-}K\left(ReLU\left(\hat{X_{i}^{\left(1\right)}}{\hat{X_{i}^{\left(1\right)}}}^{T}\right)\right)\in R^{N\times N},$$

where the top ten positive correlations are retained per node, which suppresses weak and negative correlations. This sparsification helps restrict the graph convolution range for computational efficiency while focusing on the most relevant intra-regional interactions.

#### 3.2.2. Node-Wise Temporal Embedding

To effectively capture the temporal dynamics at each node and generate task-relevant node features, a node-wise temporal embedding approach is employed, while the adjacency matrices define the spatial topology of the graph, modeling temporal patterns is also crucial for learning discriminative node features. The goal of this module is to encode the temporal signal of each node, $X_{i}^{\left(1\right)}\in R^{T}$, into a fixed-length representation, $X_{i}^{\left(2\right)}\in R^{T_{1}}$, which reduces the dimensionality while retaining essential discriminative temporal characteristics. Specific parameters are detailed in Table 3.

To achieve this, we employ temporal convolutional layers followed by a time-attention pooling mechanism. Specifically, a two-layer convolutional stack is first applied to extract local temporal features from the input signal. This step focuses on capturing important temporal dynamics within the signal, which are then enhanced by the attention mechanism. The attention mechanism assigns dynamic weights to each time step based on its relevance to the task, allowing the model to focus on the most informative segments of the signal. In this way, the model emphasizes discriminative temporal patterns that are critical for the task. The attention mechanism is defined as

(6) $$S=Softmax\left(W\cdot {\hat{H_{i}}}^{3}\right),\;{\hat{H_{i}}}^{3}=reshape\left(H_{i}^{2}\right),$$

where *W* represents the attention weights, and the softmax operation emphasizes the most salient time steps across the temporal sequence. The attention-weighted feature map is then summed across the temporal dimension:

(7) $$H_{i}^{5}=\sum_{t=1}^{T}S_{t}\cdot H_{i}^{3}\left(t\right),$$

where $H_{i}^{5}\in R^{N\times C_{2}}$ represents the final node-level feature map. Finally, the node-level representation is projected to a lower-dimensional space using a linear layer, producing the feature embedding $X_{i}^{\left(2\right)}\in R^{N\times T_{1}}$. This attention-guided temporal embedding reduces redundant temporal information while preserving critical patterns, thereby improving the discriminative capacity of node-level features for graph convolution.

#### 3.2.3. Intra-Region Graph Convolution

After generating region-wise graphs and temporal embeddings, localized graph convolution is applied to enable structured feature interactions among nodes within each region. To model inter-channel interactions efficiently, Chebyshev polynomial approximation (order-3) [35] is adopted for spectral graph convolution, enabling localized information propagation within a three-hop neighborhood.

For each region i∈{r,l} (left and right hemispheres), the normalized Laplacian is computed as

(8) $$L_{i}=I-D_{i}^{-1/2}A_{i}D_{i}^{-1/2}\in R^{N\times N},\;i\in \left\{r,l\right\}$$

where $D_{i}$ is the degree matrix of the adjacency matrix $A_{i}$.

Each graph convolution block consists of two stacked Chebyshev convolution layers with residual connections and layer normalization, followed by a ReLU activation and a dropout layer (dropout=0.2). The output of the convolution layers is given by

(9) $$\begin{array}{cc} X_{i}^{\left(3\right)} & =LN\left(X_{i}^{\left(2\right)}+Dropout\left(\sigma \left(ChebConv\left(X_{i}^{\left(2\right)}\right)\right)\right)\right),\;i\in \left\{r,l\right\} \end{array}$$

where σ(·) is the ReLU activation function, and LN(·) represents layer normalization.

Subsequently, region-level embeddings $X_{i}^{\left(4\right)}\in R^{1\times T_{2}}$ are obtained by averaging across nodes within each region:

(10) $$X_{i}^{\left(4\right)}=\frac{1}{N}\sum_{j=1}^{N}X_{i}^{\left(3\right)}\left(j,:\right)$$

This pooling operation aggregates information from all nodes in the region, producing a fixed-length representation that captures the region-specific dynamics. The intra-region graph convolution mechanism refines node embeddings by local interactions within the region, allowing the model to capture complex spatial and temporal patterns, enhancing the model’s ability to learn discriminative features.

### 3.3. Attentive Fusion Classification Module

The attentive fusion classification (AFC) module is designed to adaptively aggregate region-level features and fuse information across regions, enhancing the model’s ability to capture both intra-region and inter-region dependencies. While intra-region features capture local dependencies within each brain region, the AFC module focuses on aggregating and fusing these region-specific features to model inter-region interactions, which are essential for more comprehensive feature representation.

To achieve this, the region-level embeddings $X_{r}^{\left(4\right)}$ and $X_{l}^{\left(4\right)}$ from the right and left hemispheres are concatenated to form $X^{\left(5\right)}\in R^{2\times T_{2}}$. An attention mechanism is then applied to estimate the importance of each region, using a linear transformation followed by softmax to compute the attention weights $w\in R^{2\times 1}$. Furthermore, the final feature vector $X^{\left(6\right)}$ is obtained by performing a weighted sum of the region embeddings using the attention weights:

(11) $$X^{\left(6\right)}=w^{T}X^{\left(5\right)}\in R^{1\times T_{2}}$$

Finally, the fused vector is normalized and passed through a fully connected layer for MDD classification. The attention-based fusion mechanism enables the model to dynamically emphasize the most relevant inter-region dependencies, resulting in more robust and interpretable predictions.

## 4. Experimental Results and Discussion

### 4.1. Experimental Setup

**Figure 4:**
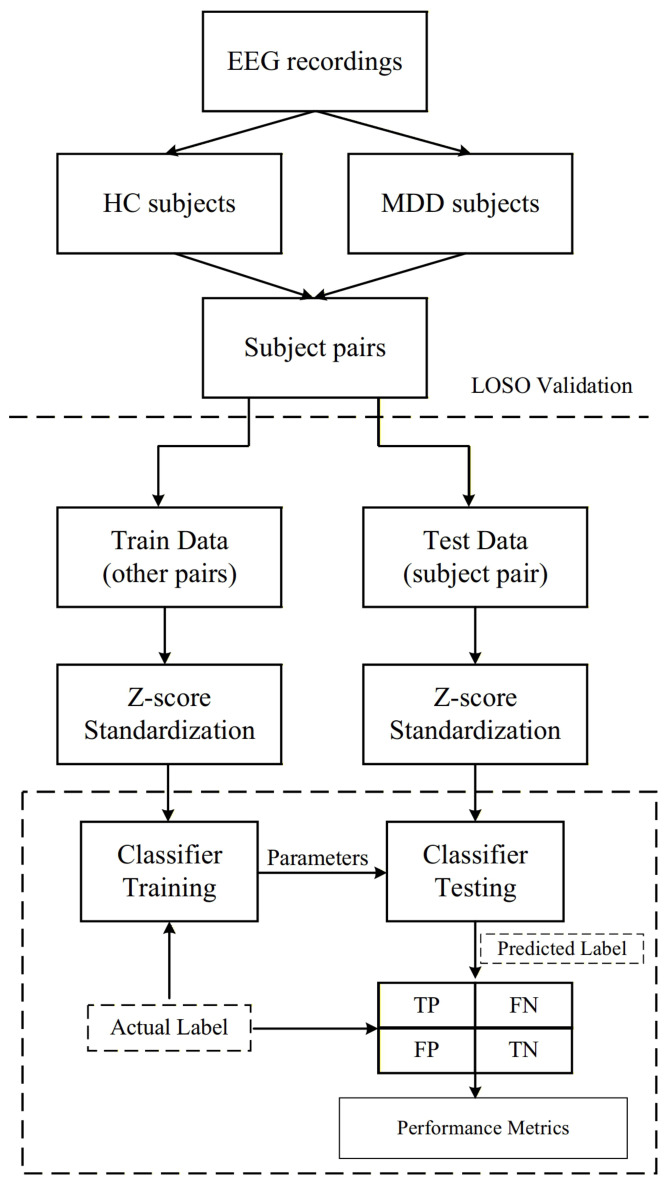
Experimental procedure.

In this study, we conduct a binary classification task (MDD vs. HC) on the private dataset to evaluate the effectiveness and robustness of the proposed MSBP-GNN under a cross-subject scenario, which better reflects real-world deployment where unseen subjects are encountered. As shown in Figure 4, we form subject pairs by combining one MDD subject and one NC subject, and adopt a leave-one-pair-out protocol. In each iteration, all samples from one subject pair are held out as the test set, while samples from the remaining subject pairs are used for model training. From the training portion, we further split 20% of the samples as a validation set and use the remaining 80% for training. The model is trained for 50 epochs, and the checkpoint with the best validation performance is selected for evaluation on the held-out test pair. For each recording corresponding to a particular state (EC or EO) of a subject, we uniformly selected the middle 180 s of EEG signals for analysis. The samples were split using a 2-s non-overlapping sliding window from the raw EEG signals.

All experiments are performed on a server equipped with a 4.20GHz Intel(R) Core(TM) i7-7700K CPU, an NVIDIA GeForce GTX TITANX GPU, and 16GB RAM. The model is optimized using Adam with an initial learning rate of 0.001. A cosine annealing scheduler is adopted to adjust the learning rate during training, with $T_{\max}=50$ and $\eta_{\min}=10^{-6}$. We train for at most 50 epochs with a batch size of 64, and early stopping is applied if the validation loss does not improve for 10 consecutive epochs. Model performance on the held-out test set is evaluated using four metrics: accuracy, sensitivity, specificity, and F-measure.

### 4.2. Performance Metrics

Four performance metrics, including accuracy, sensitivity, specificity, and F-measure, are introduced to evaluate the model’s performance on the testing set. They are defined as follows:

(12) $$\mathrm{Accuracy}=\frac{TP+TN}{TP+TN+FP+FN}\cdot 100\%$$

(13) $$\mathrm{Sensitivity}\;=\frac{TP}{TP+FN}\cdot 100\%$$

(14) $$\mathrm{Specificity}\;=\frac{TN}{TN+FP}\cdot 100\%$$

(15) $$F\_measure=\frac{2\times TP}{FP+FN+2\times TP}$$

where *TP*, *TN*, *FP* and *FN* mean “true positive”, “true negative”, “false positive” and “false negative”, respectively. Among them, F-measure can be regarded as a weighted harmonic average of sensitivity and specificity.

### 4.3. Related Methods for Comparison

In order to demonstrate the effectiveness of the proposed MSBP-GNN framework, we reproduce five representative methods under the same experimental setting and compare them with our method. For fair comparison, all baseline models were trained from scratch under the same dataset, preprocessing procedure, cross-subject split, and training/validation/testing framework as the proposed model. The brief introduction of the five comparison methods is as follows.

(1) LMDA [36]: A lightweight attention network that incorporates a multi-dimensional attention module to enhance EEG feature modeling.
(2) DeprNet [37]: A CNN model designed for depression recognition based on EEG signals.
(3) EEGNet [38]: A compact CNN designed for EEG decoding, emphasizing efficient feature extraction and robust classification performance.
(4) MV-SDGC [39]: Extracting time–frequency and energy information, integrating these features using a GCN and attention mechanism, and then inputting them into the classification block.
(5) EEG-Conformer [40]: A hybrid model that utilizes a CNN to extract local spatiotemporal features and a Transformer to capture long-range dependencies.

### 4.4. Experimental Results

**Figure 5:**
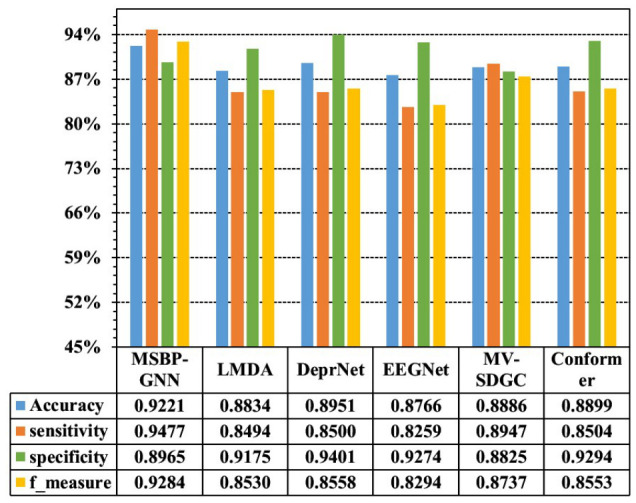
Graphical comparison of classification performance.

Figure 5 and Table 4 present the comparative results of the proposed MSBP-GNN and five baseline methods on the private dataset under the leave-one-subject-pair-out setting. Figure 5 shows the overall comparison of the average classification performance, while Table 4 further reports the mean ± standard deviation across all subject pairs and the *p*-values obtained from one-sided paired *t*-tests on fold-wise accuracy values, under the directional hypothesis that MSBP-GNN outperforms each baseline. As shown, MSBP-GNN achieves the best average overall performance among the compared methods, reaching 92.21 ± 12.81% in accuracy, 94.77 ± 11.56% in sensitivity, 89.65 ± 21.49% in specificity, and 0.9284 ± 0.1168 in F1-score. The paired statistical analysis further supports the superiority trend of MSBP-GNN over the baseline methods, with relatively smaller *p*-values observed against EEGNet and LMDA. Considering that cross-subject EEG decoding is inherently challenging due to substantial inter-subject variability, these results indicate that the proposed multi-scale brain-partition-based framework provides an effective and robust solution for cross-subject MDD detection.

**Figure 6:**
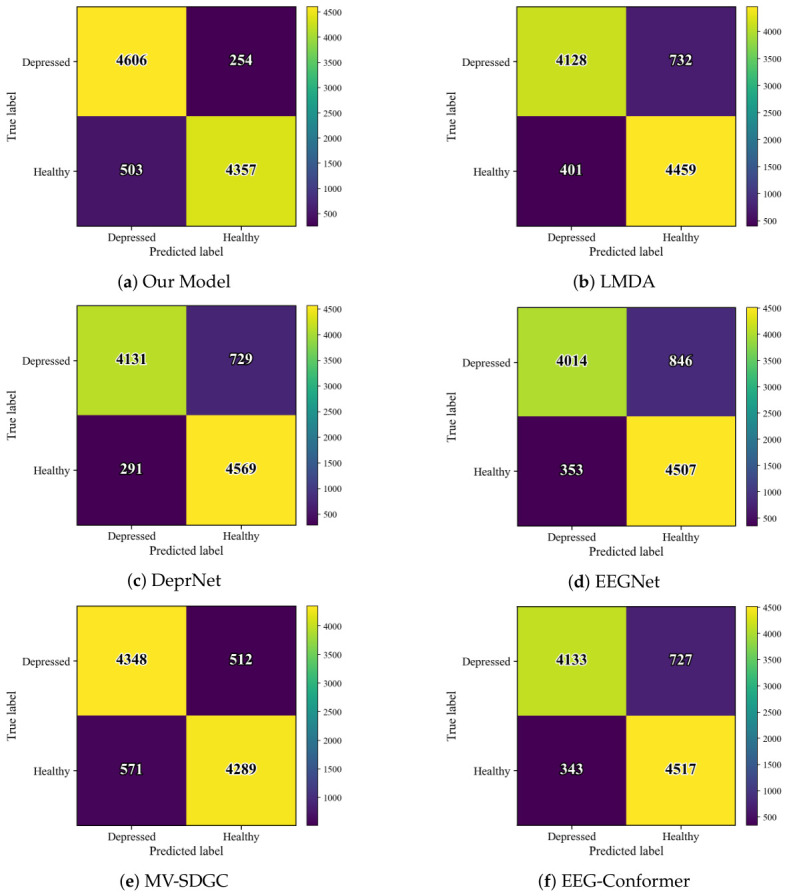
Confusion matrices for tested models.

To further evaluate diagnostic reliability at the sample level, Figure 6 shows the confusion matrices aggregated over all test subject pairs under the leave-one-subject-pair-out protocol. Among the comparison models, MSBP-GNN achieves the highest number of correctly classified samples, with 4606 true positives and 4357 true negatives. Notably, it yields the fewest false negatives (FN=254), indicating the strongest ability to identify depressed samples while maintaining a low false positive count (FP=503). LMDA produces fewer false positives (FP=401), but at the cost of a markedly increased number of false negatives (FN=732). DeprNet yields the fewest false positives (FP=291), yet also shows a relatively large number of false negatives (FN=729). EEGNet gives the highest false negative count (FN=846), suggesting weaker sensitivity to depressed samples, whereas MV-SDGC produces the highest false positive count (FP=571), indicating reduced specificity. EEG-Conformer also shows relatively high false negative and false positive counts (FN=727, FP=343). Overall, these results suggest that MSBP-GNN achieves a balance between sensitivity and specificity at the aggregated sample level, which is consistent with its best average overall performance in the cross-subject setting.

### 4.5. Ablation and Sensitivity Experiments

To further examine the contribution of the main design choices in MSBP-GNN, we conduct a series of fine-grained ablation and sensitivity experiments. Specifically, we evaluate the effects of the multi-scale temporal filtering design, the brain parcellation strategy, the region-level fusion mechanism, and the graph sparsity setting. The corresponding results are summarized in Table 5, Table 6, Table 7 and Table 8.

#### 4.5.1. Effect of Multi-Scale Temporal Filtering

To verify the effectiveness of the multi-scale temporal filtering module, we compare the full three-scale configuration with several reduced variants, including single-scale settings and different two-scale combinations. As shown in Table 5, the complete three-scale setting (0.4,0.2,0.1), which is also the default configuration of MSBP-GNN, achieves the best overall performance, with the highest accuracy, sensitivity, and F1-score.

Among the single-scale settings, (0.1) performs better than (0.2) and (0.4), suggesting that the shorter temporal receptive field is more effective than the other individual scales in the current task. The two-scale variants generally improve over the weaker single-scale settings, indicating that combining temporal information from different receptive fields is beneficial. In particular, the (0.4,0.1) combination achieves the best performance among all two-scale variants, but still remains below the full three-scale model.

Overall, these results suggest that different temporal scales provide complementary information for cross-subject MDD detection, and that the complete multi-scale design offers the most effective temporal representation in the proposed framework.

#### 4.5.2. Effect of Brain Parcellation Strategy

To evaluate the effect of brain partition priors, we compare different parcellation strategies, including the proposed two-region hemispheric partition, a four-region partition, an anterior–posterior (AP) partition, and a non-parcellation full-brain graph setting. The corresponding results are summarized in Table 6.

Among all tested strategies, the proposed two-region hemispheric partition achieves the best overall performance, yielding the highest accuracy, sensitivity, and F1-score. This result suggests that hemispheric partitioning provides an effective structural prior for organizing EEG channel interactions in the current cross-subject MDD detection setting.

In comparison, the four-region partition leads to lower overall performance, indicating that a finer spatial division does not necessarily result in more discriminative representations under the current setting. The AP partition attains the highest specificity, suggesting a relatively stronger ability to identify healthy samples, but its sensitivity and F1-score are clearly lower than those of the proposed model. The full-brain setting performs better than the four-region and AP settings on some metrics, but still underperforms the two-region model in overall detection effectiveness.

Overall, these results indicate that incorporating brain partition priors is beneficial for cross-subject EEG modeling, and that the proposed two-region hemispheric partition provides a more favorable balance between structural simplicity and discriminative performance than the alternative strategies considered here.

#### 4.5.3. Effect of Fusion Mechanism

To evaluate the effect of the region-level fusion design, we compare different fusion strategies, including attention-based fusion, average pooling, max pooling, and concatenation followed by a multilayer perceptron (MLP). The corresponding results are summarized in Table 7.

Among the compared strategies, the attention-based fusion achieves the best overall performance, yielding the highest accuracy, sensitivity, and F1-score. This suggests that different regional representations do not contribute equally to MDD detection, and that adaptive weighting is more effective than fixed fusion rules for integrating region-level information.

In comparison, max pooling achieves the highest specificity and shows the second-best overall performance, indicating that it can preserve relatively strong discriminative regional responses, but still remains inferior to the proposed attention-based fusion in terms of overall detection effectiveness. By contrast, average pooling and concatenation with MLP both lead to weaker overall performance.

Overall, these results support the use of the attention-based fusion mechanism in the final MSBP-GNN, as it provides a more effective way to integrate region-level representations in the current cross-subject setting.

#### 4.5.4. Sensitivity to Graph Sparsity

To investigate the effect of graph sparsity, we conduct a sensitivity analysis on the Top-K parameter used in graph construction. The corresponding results are summarized in Table 8.

The results show that model performance varies with the graph sparsity level. Among the tested settings, K=10 achieves the best overall performance, yielding the highest accuracy, sensitivity, and F1-score. This suggests that retaining relatively richer intra-regional connectivity is beneficial for cross-subject MDD detection in the current setting.

In comparison, smaller Top-K values generally lead to weaker overall performance. In particular, the very sparse setting K=2 achieves the highest specificity, but shows clearly lower accuracy, sensitivity, and F1-score, indicating that excessive sparsification may improve healthy-sample discrimination at the cost of reduced overall detection effectiveness. The intermediate settings K=4, K=6, and K=8 provide more balanced results, but still remain below K=10 in overall performance.

Overall, these results indicate that an appropriate graph sparsity level is important for balancing structural compactness and preservation of informative functional interactions. Based on this analysis, K=10 is adopted as the final configuration in the proposed model.

### 4.6. Discussion

The above results further demonstrate the value of introducing structured brain priors into cross-subject EEG modeling. Instead of treating all EEG channels as an undifferentiated whole, the proposed MSBP-GNN organizes them through hemispheric partitioning and region-wise graph learning, providing a more structured representation of coordinated brain activity. The comparative results on different partition strategies indicate that the two-region setting is more suitable for the current task, suggesting that hemispheric partitioning serves as an effective modeling prior for cross-subject MDD detection.

Meanwhile, the ablation results also support the complementary roles of the different modules in the proposed framework. The multi-scale temporal design enhances the model’s ability to capture EEG dynamics under different temporal receptive fields, while the attention-based fusion strategy enables adaptive integration of region-level representations. Together with partition-based graph construction, these components form a unified framework for jointly modeling temporal characteristics and structured spatial interactions in EEG signals. Overall, these findings show that the proposed design is effective not only in improving detection performance, but also in providing a more physiologically inspired modeling perspective for EEG-based depression analysis.

## 5. Conclusions

MDD severely impacts the daily lives of patients and their families and remains a prevalent mental health issue worldwide. Efficient and objective MDD detection is therefore important for timely screening and intervention. In this study, we proposed MSBP-GNN, an EEG-based framework that integrates multi-scale temporal filtering, brain-partition-based graph learning, and attention-based fusion for cross-subject MDD detection. By jointly modeling temporal dynamics and structured spatial interactions, the proposed method is able to capture both intra-region and inter-region dependencies in brain activity. Experimental results on the private dataset demonstrate that MSBP-GNN achieves favorable overall performance, and the ablation and sensitivity experiments further verify the effectiveness of its main design choices. These findings suggest that incorporating structured brain priors into graph-based EEG modeling is beneficial for cross-subject MDD detection. Nevertheless, this study is based on a private dataset from a single center, and the current task focuses on MDD detection. In future work, we will further improve cross-subject robustness and generalizability, expand the dataset scale, and explore more advanced feature modeling strategies for clinically oriented EEG-based depression analysis.

## Figures and Tables

**Table 1:** Age and sex distribution of subjects. The between-group comparison of overall age showed no significant difference (p=0.289).

Diagnosis	Female	Female Age (Mean ± Std)	Male	Male Age (Mean ± Std)	Total	Overall Age (Mean ± Std)
Healthy	14	35.36 ± 4.78	13	36.08 ± 4.31	27	35.70 ± 4.49
MDD	12	33.50 ± 3.90	15	40.07 ± 4.59	27	37.15 ± 5.37

**Table 2:** The specific layers and parameter settings of the multi-scale temporal filtering module.

Layers	Details	Output Sizes
${DWConv}_{k_{1}}$	$C@k_{1}\left(C\right)$	C×T
*BN*	ELU	$X_{1}:C\times T$
${DWConv}_{k_{2}}$	$C@k_{2}\left(C\right)$	C×T
*BN*	ELU	$X_{2}:C\times T$
${DWConv}_{k_{3}}$	$C@k_{3}\left(C\right)$	C×T
*BN*	ELU	$X_{3}:C\times T$
stack	$X_{1},X_{2},X_{3}$	3×C×T
mean	dim=0	$X^{\left(1\right)}:C\times T$

D@K(J) means *D* kernels with a size of *K* and the number of groups is *J*. In this work, the parameters are set as follows: C=19, T=512, $k_{1}=103$, $k_{2}=51$, $k_{3}=25$ (note that the kernel size is adjusted to odd values for same padding).

**Table 3:** The specific layers and parameter setting of node-wise temporal embedding block.

Layers	Details	Output Sizes
unsqueeze	dim = 1	$H_{i}:N\times 1\times T$
Conv2d_1_	$NC_{1}@1\times 7\left(N\right)$	$NC_{1}\times 1\times T$
*ReLU*		$H_{i}^{1}:N\times C_{1}\times T$
Conv2d_2_	$NC_{2}@C_{1}\times 7\left(N\right)$	$NC_{2}\times 1\times T$
*ReLU*		$H_{i}^{2}:NC_{2}\times 1\times T$
reshape		$H_{i}^{3}:N\times C_{2}\times T$
Conv2d_*p*_	$N@C_{2}\times 1$	S:N×1×T
Softmax	S,dim = 2	W:1×T
	$S\times H_{i}^{3}$	$H_{i}^{4}:N\times C_{2}\times T$
sum	dim = 2	$H_{i}^{5}:N\times C_{2}$
Linear	($C_{2}$, $T_{1}$)	$X_{i}^{\left(2\right)}:N\times T_{1}$

D@K(J) means *D* kernels with size of *K* and the number of groups is *J*. In this work, the parameters are set as follows: N=11, $C_{1}=16$, $C_{2}=32$, $T_{1}=64$.

**Table 4:** Classification results of our work and comparison works in cross-subject scenario.

Model/Dataset	Accuracy (%)	Sensitivity (%)	Specificity (%)	*F_measure*	*p*-Value
MSBP-GNN	92.21 ± 12.81	94.77 ± 11.56	89.65 ± 21.49	0.9284 ± 0.1168	–
LMDA	88.34 ± 15.65	84.94 ± 27.58	91.75 ± 20.61	0.8530 ± 0.2357	0.0790
DeprNet	89.51 ± 16.37	85.00 ± 29.31	94.01 ± 18.92	0.8558 ± 0.2702	0.1939
EEGNet	87.66 ± 17.28	82.59 ± 31.75	92.74 ± 20.34	0.8294 ± 0.2825	0.0778
MV-SDGC	88.86 ± 15.84	89.47 ± 24.10	88.25 ± 25.57	0.8737 ± 0.2194	0.1055
Conformer	88.99 ± 17.01	85.04 ± 29.21	92.94 ± 21.41	0.8553 ± 0.2556	0.1411

The reported values are mean ± standard deviation across all subject pairs. The *p*-values are computed using one-sided paired *t*-tests on fold-wise accuracy values between MSBP-GNN and each baseline.

**Table 5:** Fine-grained ablation study of the multi-scale temporal filtering module under different temporal scale combinations. The reported values are mean ± standard deviation across all subject pairs.

Temporal Scales	Accuracy (%)	Sensitivity (%)	Specificity (%)	F1-Score
(0.4)	83.15 ± 20.69	81.38 ± 31.44	84.92 ± 28.51	0.7978 ± 0.2839
(0.2)	85.64 ± 17.27	82.02 ± 30.49	89.26 ± 22.68	0.8172 ± 0.2650
(0.1)	88.76 ± 15.96	86.48 ± 24.01	91.03 ± 20.77	0.8707 ± 0.2137
(0.4,0.2)	88.02 ± 16.47	86.01 ± 26.39	90.04 ± 22.70	0.8558 ± 0.2344
(0.4,0.1)	89.56 ± 13.82	87.63 ± 20.72	91.48 ± 22.96	0.8864 ± 0.1526
(0.2,0.1)	84.24 ± 18.97	80.27 ± 30.95	88.21 ± 27.04	0.8036 ± 0.2765
(0.4,0.2,0.1)	92.21 ± 12.81	94.77 ± 11.56	89.65 ± 21.49	0.9284 ± 0.1168

**Table 6:** Comparison of different brain parcellation strategies. The reported values are mean ± standard deviation across all subject pairs.

Parcellation Strategy	Accuracy (%)	Sensitivity (%)	Specificity (%)	F1-Score
Two-region (MSBP-GNN)	92.21 ± 12.81	94.77 ± 11.56	89.65 ± 21.49	0.9284 ± 0.1168
Four-region	86.10 ± 16.64	84.81 ± 26.72	87.39 ± 24.36	0.8371 ± 0.2380
AP partition	87.08 ± 17.92	81.95 ± 30.32	92.20 ± 25.25	0.8297 ± 0.2769
Full-brain	88.54 ± 15.53	85.84 ± 25.52	91.23 ± 23.73	0.8627 ± 0.2166

**Table 7:** Comparison of different region-level fusion strategies. The reported values are mean ± standard deviation across all subject pairs.

Fusion Strategy	Accuracy (%)	Sensitivity (%)	Specificity (%)	F1-Score
attn	92.21 ± 12.81	94.77 ± 11.56	89.65 ± 21.49	0.9284 ± 0.1168
avg	87.13 ± 16.92	82.67 ± 30.87	91.58 ± 21.79	0.8278 ± 0.2761
max	91.46 ± 14.34	88.70 ± 24.69	94.22 ± 17.89	0.8899 ± 0.2249
concat_mlp	85.67 ± 18.12	81.23 ± 29.85	90.10 ± 22.28	0.8191 ± 0.2656

**Table 8:** Sensitivity analysis of the Top-K parameter in graph construction. The reported values are mean ± standard deviation across all subject pairs.

Top-K	Accuracy (%)	Sensitivity (%)	Specificity (%)	F1-Score
2	87.74 ± 16.51	84.28 ± 29.70	91.19 ± 21.27	0.8385 ± 0.2725
4	88.93 ± 14.40	86.71 ± 20.55	91.15 ± 25.10	0.8813 ± 0.1531
6	89.10 ± 12.63	89.26 ± 19.82	88.95 ± 20.76	0.8846 ± 0.1420
8	89.13 ± 15.06	89.07 ± 22.35	89.18 ± 24.83	0.8781 ± 0.2083
10	92.21 ± 12.81	94.77 ± 11.56	89.65 ± 21.49	0.9284 ± 0.1168

## Data Availability

The data used in this study is a private dataset.
